# Predictors of metabolic abnormalities in phenotypes that combined anthropometric indices and triglycerides

**DOI:** 10.1186/s12906-016-1024-1

**Published:** 2016-02-10

**Authors:** Bum Ju Lee, Jiho Nam, Jong Yeol Kim

**Affiliations:** KM Fundamental Research Division, Korea Institute of Oriental Medicine, 1672 Yuseongdae-ro, 1672 Yuseongdae-ro, Yuseong-gu, Deajeon 305-811 Republic of Korea

**Keywords:** Hypertriglyceridemic waist phenotype, Predictor, Machine learning, Anthropometry, Metabolic abnormalities, Triglycerides

## Abstract

**Background:**

The hypertriglyceridemic waist (HW) phenotype has been shown to be strongly associated with metabolic abnormalities; however, to date, no study has reported the prediction of metabolic abnormalities using the HW phenotype along with waist circumference (WC) and the triglyceride (TG) level or various phenotypes consisting of an individual anthropometric index combined with the TG level. The objectives of this study were to assess the association of the HW phenotype with metabolic abnormalities in Korean women and to evaluate the predictive powers of various phenotypes with regard to metabolic abnormalities.

**Methods:**

Total cholesterol (TC), high- and low-density lipoprotein (HDL and LDL) cholesterol, and TG levels, systolic and diastolic blood pressures (SBP and DBP), and anthropometric indices were measured in 7661 women. The Naive Bayes algorithm and logistic regression were used to determine the predictive powers of the models using different phenotypes.

**Results:**

The HW phenotype demonstrated the strongest association with all metabolic components. The best phenotypic predictors were the forehead-to-rib circumference ratio + TG for the HDL level, age + TG for the LDL level, age + TG for SBP, and rib circumference + TG and neck circumference + TG for DBP. The associations between TG and TC or HDL were higher compared with those between WC and TC or HDL, whereas the associations between WC and SBP or DBP were higher compared with those between TG and SBP or DBP. Age was strongly associated with hypercholesterolemia, the HDL and LDL cholesterol levels, and SBP and had good predictive power, but not with respect to DBP.

**Conclusions:**

We have determined that the HW phenotype is a useful indicator of metabolic abnormalities in Korean women; although HW had the strongest association with metabolic abnormalities, the best phenotype combination consisting of a single anthropometric index and the TG level may differ depending on the metabolic factors in question. Our findings provide insights into the detection of metabolic abnormalities in complementary and alternative medicine.

## Background

In recent years, there have been many attempts to determine the clinical importance of the hypertriglyceridemic waist (HW) phenotype in various diseases, such as cardiovascular diseases (CVDs) and metabolic abnormalities, because there is a critical need for convenient, easy and cost-effective tools to evaluate risk factors for these conditions [1]. The HW phenotype has been defined as a combination of enlarged waist circumference (WC) and a high plasma triglyceride (TG) level. Previous studies have indicated that the HW phenotype is closely associated with metabolic abnormalities [1–15], diabetes mellitus [3, 7, 9, 16–24], CVD [5, 6, 8, 9, 14, 25–32], and chronic kidney disease [33]. Furthermore, accumulating evidence suggests that this phenotype is a useful indicator of several diseases.

Numerous studies have demonstrated that the HW phenotype is strongly associated with components of metabolic abnormalities and is a strong indicator of these abnormalities [1–15]. For example, Arsenault et al. [2] found that subjects with the HW phenotype had smaller low-density lipoprotein (LDL) particles, higher blood pressure, lower levels of apolipoprotein A-I and high-density lipoprotein (HDL) cholesterol, and higher levels of apolipoprotein B and C-reactive protein compared with subjects with a normal WC and TG level. Amini et al. [3] found that men with the HW phenotype had higher TG and cholesterol levels and a lower HDL level compared with men without HW or with a high WC or elevated TG level. They also found that women with HW had higher fasting blood sugar, 2 h-post prandial blood sugar, hemoglobin, TG, cholesterol, and LDL levels and a lower HDL level compared with women without HW or with a high WC or elevated TG level. Esmaillzadeh et al. [4] indicated that adolescents with HW had a strong tendency to have risk factors for hypercholesterolemia and high LDL cholesterol and low HDL cholesterol levels and that HW was a stronger predictor compared with the TG level, WC, and weight. Hiura et al. [5] reported that HW was associated with higher total cholesterol (TC), LDL, and TG levels and an increased TC-to-HDL ratio and that WC alone could not predict CVD risk. Alavian et al. [1] suggested that HW was a useful indicator of metabolic risk in children and was suitable for population-based studies. de Graaf et al. [6] examined the utility of HW for the identification of coronary artery disease evaluated by computed tomographic coronary angiography (CTA) in men and women with type-2 diabetes; these authors determined that HW was strongly associated with the inflammatory profile, TG, TC, HDL and LDL cholesterol levels, and coronary artery disease. Yu et al. [7] suggested the following cutoff values for HW to predict type-2 diabetes in Chinese adults: WC ≥ 75 cm and TG ≥ 110 mg/dl. Czernichow et al. [8] concluded from a 7.5-year longitudinal study that HW was a good predictor of CVD in middle-aged French men. StPierre et al. [9] documented that Canadian men and women with type-2 diabetes and HW exhibited their first CVD manifestations 5 years earlier compared with individuals without HW; furthermore, they reported that HW was an important predictor of CVD symptoms.

These studies have suggested that the HW phenotype is a risk factor for metabolic abnormalities and that this phenotype can be used as a primary screening tool for public health; however, to date, no study has compared the predictive powers of the HW phenotype combined with the TG level and WC and phenotypic combinations of individual anthropometric indices with the TG level for the identification of metabolic abnormalities. The objectives of this study were to assess the associations of the HW phenotype with metabolic factors, such as the TC, HDL and LDL levels, and systolic and diastolic blood pressures (SBP and DBP, respectively) in Korean women and to evaluate the powers of the HW phenotype combined with the TG level and WC and of various phenotypes consisting of single anthropometric indices and the TG level to predict metabolic abnormalities. The evaluations of all predictive models were based on two machine-learning algorithms. Additionally, this study aimed to compare whether phenotypes consisting of single indices and the TG level were better in terms of the ability to predict metabolic abnormalities. This study may aid in the development of decision support systems for the initial screening of metabolic abnormalities in women. To our knowledge, this is the first study evaluating the predictive powers of phenotypes consisting of the TG level and single anthropometric indices to identify individuals with metabolic abnormalities.

## Methods

### Participants

The data used in this retrospective cross sectional study were provided by the Korean Health and Genome Epidemiology Study database (KHGES). All participants were recruited from hospitals in Anseong, Ansan, and other cities in the Republic of Korea between November 2006 and August 2013. A total of 7661 Korean women aged 20–85 years participated in this study. The Korea Institute of Oriental Medicine (KIOM) Institutional Review Board approved the study, and written informed consent was obtained from all participants.

### Measurements and data collection

We collected subjects’ demographic and clinical characteristics from self-reported data. TC, HDL, LDL, SBP, and DBP levels were measured for all participants by trained clinicians based on standardized protocols (ADVIA1800, Siemens, USA), and all participants were required to fast for at least 8 h prior to testing. BP was measured manually from the left upper arm after a sufficient period of rest using a standard sphygmomanometer.

Anthropometric indices were estimated by trained observers or clinicians using standard operating procedures. Height and weight were measured to the nearest 0.1 cm and 0.1 kg, respectively, for subjects who wore lightweight clothing without shoes (LG-150; G Tech International Co., Ltd., Uijeongbu, Republic of Korea). The circumferences of the forehead, neck, axilla, chest, rib, waist, pelvis, and hip were estimated using non-elastic tape. We then computed ratio indices, such as the waist-to-hip ratio (WHR) and body mass index (BMI), which are widely used in medicine [34, 35]. Detailed information on the measurement positions and protocols for humans has been described in several previous studies [34–37]. In brief, forehead circumference (ForeheadC) was gauged at the levels of the glabella and occiput of the head. Neck (NeckC) and axillary circumferences (AxillaryC) were measured at the levels of the thyroid cartilage and cricoid cartilage and at the levels of the left and right axillae, respectively. Chest (ChestC) and rib circumferences (RibC) were measured at the levels of the left and right nipples and at the levels of the left and right 7th and 8th prominences of the costochondral junction, respectively. Waist circumference (WaistC, WC) was gauged at the level of the umbilicus. Further, pelvic (PelvicC) and hip circumferences (HipC) were measured at the levels of the left and right anterior superior iliac spines and at the level of the upper edge of the pubis, respectively [34–37].

The inclusion criteria of the subjects were as follows: 1) submission of written informed consent; 2) women aged 20-85 years; and 3) Koreans residing in the Republic of Korea. The exclusion criteria were as follows: 1) omission of blood information; and 2) omission of anthropometric measurements.

### Definitions

The diagnosis of metabolic abnormalities was based on the recommendations of the National Cholesterol Education Program Adult Treatment Panel III (NCEP-ATP III) [38]. Hypercholesterolemia was defined as TC ≥ 240 mg/dL; hypo-HDL cholesterolemia was defined as HDL < 50 mg/dL; and hyper-LDL cholesterolemia was defined as LDL ≥ 160 mg/dL. Hypertension was defined as SBP ≥ 130 mmHg and/or DBP ≥ 85 mmHg. The HW phenotype was defined as WC ≥ 85 cm and TG ≥ 1.5 mmol/L (133 mg/dl), in accordance with the criteria used for women in previous studies [2, 30, 32, 39]. Table 1 lists all features related to the metabolic factors and anthropometric indices used in this study.

**Table 1:** All features related to metabolic components and anthropometric indices in Korean women (Std.: standard deviation)

Variable	Mean (Std.)	Description
Subjects	7661	Number of female subjects
HW subjects	1484	Number of subjects with the HW phenotype
TC (mg/dL)	192.1 (34.78)	Total cholesterol
HDL (mg/dL)	50.65 (13.36)	High-density lipoprotein cholesterol
LDL (mg/dL)	116.1 (32.26)	Low-density lipoprotein cholesterol
TG	121.2 (73.04)	Triglycerides
SBP (mmHg)	118.6 (16.74)	Systolic blood pressure
DBP (mmHg)	76.64 (10.67)	Diastolic blood pressure
Height	155.9 (6.17)	Height
Weight	57.82 (8.435)	Weight
BMI	23.81 (3.358)	Body mass index
Age	53.03 (13.76)	Age
NeckC	33.28 (2.214)	Neck circumference
ChestC	90.47 (8.017)	Chest circumference
RibC	79.64 (8.263)	Rib circumference
WaistC (WC)	83.99 (9.306)	Waist circumference
HipC	92.95 (6.049)	Hip circumference
Neck_Hip	0.359 (0.022)	Neck-to-hip circumference ratio
Axillary_Hip	0.945 (0.047)	Axillary-to-hip circumference ratio
Chest_Hip	0.973 (0.06)	Chest-to-hip circumference ratio
Rib_Hip	0.856 (0.064)	Rib-to-hip circumference ratio
Waist_Hip (WHR)	0.903 (0.072)	Waist-to-hip circumference ratio
Pelvic_Hip	0.973 (0.042)	Pelvic-to-hip circumference ratio
Forehead_Waist	0.662 (0.073)	Forehead-to-waist circumference ratio
Neck_Waist	0.399 (0.034)	Neck-to-waist circumference ratio
Forehead_Rib	0.697 (0.071)	Forehead-to-rib circumference ratio
Forehead_Chest	0.612 (0.053)	Forehead-to-chest circumference ratio
WHtR	0.371 (0.051)	Waist-to-height circumference ratio

### Statistical analysis

Statistical analyses were performed with SPSS 19 for Windows (SPSS Inc., Chicago, IL, USA). Crude and age- and site-adjusted analyses were conducted using binary logistic regression (LR) to test for differences between the subjects with metabolic abnormalities and normal subjects regarding the HW phenotype and each individual index after standardization (odds ratios (ORs) and 95 % confidence intervals [CIs]). All predictive analyses were performed using the Waikato Environment for Knowledge Analysis (WEKA) data mining tool. For a more reliable prediction of metabolic abnormalities, two machine-learning algorithms, the Naïve Bayes algorithm (NB) and LR, were used to determine the predictive powers of all phenotypes, using combinations of individual anthropometric indices and the TG level, for the diagnosis of metabolic abnormalities. In brief, in accordance with previous studies [37, 40, 41], for a given feature (x) and label y in NB, a joint probability is estimated from the training data. Hence, it is a generative model. In contrast, LR estimates the probability(y/x) directly from the training data by minimizing error. Hence, it is a discriminative model. In addition, NB assumes that all features are conditionally independent. Thus, if some of the features are dependent on each other in the case of a large feature space, the predictive power might be low, but the LR model may work well, even if some of the variables are correlated [37, 40, 41]. We used the area under the receiver operating characteristic curve (AUC) to evaluate the predictive powers of all models. All predictions were tested using 10-fold cross-validation. Furthermore, for comparison of AUCs between a single anthropometric index and phenotype, we used the non-parametric test suggested by Delong et al. [42].

## Results

Out of a total of 7661 subjects, 1961 had metabolic syndrome (MS) according to the criteria for MS recommended by the NCEP-ATP III. With regard to each metabolic abnormality, 1258 women with the HW phenotype were in the non-hypercholesterolemia group (*n* = 6973), and 226 were in the hypercholesterolemia group (*n* = 688). In addition, 311 women with the HW phenotype were in the non-hypo-HDL cholesterolemia group (*n* = 3654), and 1173 were in the hypo-HDL cholesterolemia group (*n* = 4007). Further, 1268 women with the HW phenotype were in the non-hyper-LDL cholesterolemia group (*n* = 6918), and 216 were in the hyper-LDL cholesterolemia group (*n* = 743). A total of 1182 women with the HW phenotype were in the normal SBP group (*n* = 6756), and 302 were in the high SBP group (*n* = 905). Finally, 1163 women with the HW phenotype were in the normal DBP group (*n* = 6644), and 321 were in the high DBP group (*n* = 1017).

### TC

Table 2 lists the results of the association between the TC level and HW phenotype and the predictive powers of the models using each index/TG combination to identify hypercholesterolemia. The HW phenotype showed the strongest association with hypercholesterolemia, even after adjusting for site and age (*p* < 0.0001, OR = 2.222 [95 % CI 1.874-2.635]), adjusted OR = 1.875 [1.567-2.243]). Age (*p* < 0.0001, OR = 1.568 [1.439-1.708], AUC by NB = 0.648, AUC by LR = 0.62) and TG (*p* < 0.0001, OR = 1.528 [1.436-1.626], adjusted OR = 1.452 [1.362-1.549], AUC by NB = 0.647, AUC by LR = 0.686) were the most strongly associated with hypercholesterolemia and were strong predictors of hypercholesterolemia after the HW phenotype. A comparison of WC and the TG level as HW phenotype components revealed that the association of TG with hypercholesterolemia was higher compared with that of WC with hypercholesterolemia and that TG had greater predictive power compared with WC. Among the combinations of single variables and TG, the age + TG phenotype was the best predictor of hypercholesterolemia according to the NB results (AUC by NB = 0.699); however, NeckC + TG was the best predictor according to the LR results (AUC by LR = 0.686).

**Table 2:** Analysis of the associations and powers of models of the HW phenotype and all phenotypes consisting of an individual index with/without the TG level to predict hypercholesterolemia

Variable	Crude		Adjustment		AUC		Phenotype	AUC	
	p	OR (95 % CI)	p	OR (95 % CI)	NB	LR		NB	LR
HW phenotype^a^	<0.0001	2.222 (1.874–2.635)	<0.0001	1.875 (1.567–2.243)					
TG	<0.0001	1.528 (1.436–1.626)	<0.0001	1.452 (1.362–1.549)	0.647	0.686			
Age	<0.0001	1.568 (1.439–1.708)			0.648	0.62	Age + TG	0.699	0.681^***^
Weight	0.0001	1.169 (1.084–1.261)	0.0001	1.177 (1.088–1.273)	0.551	0.552	Weight + TG	0.646	0.682^***^
BMI	<0.0001	1.28 (1.188–1.379)	0.0002	1.166 (1.074–1.265)	0.582	0.583	BMI + TG	0.652	0.679^***^
NeckC	0.0146	1.1 (1.019–1.187)	0.0143	1.109 (1.021–1.206)	0.53	0.531	NeckC + TG	0.638	0.686^***^
ChestC	<0.0001	1.288 (1.193–1.39)	<0.0001	1.23 (1.13–1.338)	0.576	0.578	ChestC + TG	0.644	0.677^***^
RibC	<0.0001	1.263 (1.171–1.362)	0.0004	1.172 (1.074–1.279)	0.574	0.575	RibC + TG	0.649	0.679^***^
WaistC (WC)	<0.0001	1.279 (1.184–1.381)	0.0011	1.156 (1.06–1.262)	0.574	0.575	WaistC + TG	0.647	0.679^***^
HipC	0.0207	1.095 (1.014–1.182)	0.0264	1.096 (1.011–1.188)	0.524	0.527	HipC + TG	0.648	0.684^***^
Neck_Hip	0.8888	1.006 (0.93–1.087)	0.8403	1.009 (0.927–1.097)	0.485	0.467	Neck_Hip + TG	0.64	0.684^***^
Axillary_Hip	<0.0001	1.219 (1.128–1.316)	0.0001	1.186 (1.091–1.289)	0.554	0.557	Axillary_Hip + TG	0.635	0.676^***^
Chest_Hip	<0.0001	1.313 (1.216–1.419)	<0.0001	1.225 (1.123–1.336)	0.582	0.583	Chest_Hip + TG	0.642	0.675^***^
Rib_Hip	<0.0001	1.288 (1.192–1.391)	0.0020	1.161 (1.056–1.277)	0.573	0.576	Rib_Hip + TG	0.643	0.677^***^
Waist_Hip (WHR)	<0.0001	1.319 (1.22–1.426)	0.0046	1.145 (1.043–1.258)	0.578	0.582	Waist_Hip + TG	0.643	0.676^***^
Pelvic_Hip	<0.0001	1.328 (1.225–1.439)	0.0608	1.093 (0.996–1.2)	0.578	0.58	Pelvic_Hip + TG	0.644	0.676^***^
Forehead_Waist	<0.0001	0.736 (0.678–0.8)	0.0006	0.846 (0.768–0.931)	0.586	0.584	Forehead_Waist + TG	0.649	0.675^***^
Neck_Waist	<0.0001	0.759 (0.699–0.824)	0.0085	0.885 (0.808–0.969)	0.574	0.576	Neck_Waist + TG	0.648	0.677^***^
Forehead_Rib	<0.0001	0.742 (0.685–0.805)	0.0002	0.832 (0.755–0.916)	0.588	0.584	Forehead_Rib + TG	0.651	0.675^***^
Forehead_Chest	<0.0001	0.733 (0.676–0.795)	<0.0001	0.803 (0.733–0.88)	0.587	0.588	Forehead_Chest + TG	0.649	0.675^***^
WHtR	<0.0001	1.233 (1.144–1.329)	0.0001	1.177 (1.087–1.274)	0.569	0.57	WHtR + TG	0.648	0.68^***^

Adjustments for age and site (investigation site), *OR* odds ratio, *CI* confidence interval, *AUC* area under the receiver operating characteristic curve, *NB* Naïve Bayes, *LR* logistic regression

Significant difference between a single anthropometric index and phenotype on AUCs determined by LR, ^***^ *p* = <0.00001

^a^ There were 1258 women with the HW phenotype in the non-hypercholesterolemia group (*n* = 6973) and 226 in the hypercholesterolemia group (*n* = 688)

### HDL and LDL

Tables 3 and 4 show the associations of the HW phenotype with the HDL and LDL levels, as well as the powers of the models of all phenotypes consisting of an individual index and the TG level, to predict hypo-HDL cholesterolemia and hyper-LDL cholesterolemia. The HW phenotype was the most strongly associated with the HDL level (*p* < 0.0001, OR = 4.449 [3.889-5.09], adjusted OR = 3.646 [3.157-4.21]). The TG level showed the strongest association with the HDL level after the HW phenotype (*p* < 0.0001, OR = 2.787 [2.59-2.999], adjusted OR = 2.612 [2.415-2.825]). Among the anthropometric indices, RibC was the most strongly associated with HDL, after adjustments for site and age. The TG level (*p* < 0.0001, OR = 2.787 [2.59-2.999], adjusted OR = 2.612 [2.415-2.825]) was more strongly associated with the HDL level than with WC (*p* < 0.0001, OR = 1.737 [1.653-1.826], adjusted OR = 1.515 [1.432-1.602]). The Forehead_Rib + TG phenotype was the strongest predictor of HDL (AUC by NB = 0.722, AUC by LR = 0.73).

**Table 3:** Analysis of the associations and powers of models of the HW phenotype and all phenotypes consisting of an individual index with/without the TG level to predict hypo-HDL cholesterolemia

Variable	Crude		Adjustment		AUC		Phenotype	AUC	
	p	OR (95 % CI)	p	OR (95 % CI)	NB	LR		NB	LR
HW phenotype^a^	<0.0001	4.449 (3.889–5.09)	<0.0001	3.646 (3.157–4.21)					
TG	<0.0001	2.787 (2.59–2.999)	<0.0001	2.612 (2.415–2.825)	0.679	0.71			
Age	<0.0001	1.729 (1.646–1.815)			0.649	0.649	Age + TG	0.712	0.724^***^
Weight	<0.0001	1.33 (1.27–1.394)	<0.0001	1.396 (1.327–1.469)	0.583	0.583	Weight + TG	0.69	0.714^***^
BMI	<0.0001	1.634 (1.555–1.717)	<0.0001	1.466 (1.389–1.547)	0.64	0.64	BMI + TG	0.708	0.722^***^
NeckC	<0.0001	1.579 (1.503–1.659)	<0.0001	1.576 (1.492–1.665)	0.624	0.624	NeckC + TG	0.701	0.721^***^
ChestC	<0.0001	1.738 (1.654–1.827)	<0.0001	1.529 (1.447–1.614)	0.654	0.654	ChestC + TG	0.714	0.726^***^
RibC	<0.0001	1.84 (1.749–1.937)	<0.0001	1.603 (1.514–1.699)	0.669	0.669	RibC + TG	0.719	0.729^***^
WaistC (WC)	<0.0001	1.737 (1.653–1.826)	<0.0001	1.515 (1.432–1.602)	0.652	0.652	WaistC + TG	0.712	0.725^***^
HipC	<0.0001	1.279 (1.221–1.339)	<0.0001	1.336 (1.269–1.407)	0.564	0.565	HipC + TG	0.687	0.713^***^
Neck_Hip	<0.0001	1.242 (1.185–1.3)	<0.0001	1.169 (1.11–1.231)	0.561	0.562	Neck_Hip + TG	0.683	0.712^***^
Axillary_Hip	<0.0001	1.393 (1.329–1.46)	<0.0001	1.201 (1.14–1.264)	0.597	0.597	Axillary_Hip + TG	0.69	0.714^***^
Chest_Hip	<0.0001	1.692 (1.611–1.778)	<0.0001	1.341 (1.269–1.417)	0.646	0.646	Chest_Hip + TG	0.71	0.725^***^
Rib_Hip	<0.0001	1.844 (1.753–1.941)	<0.0001	1.502 (1.413–1.597)	0.667	0.668	Rib_Hip + TG	0.718	0.729^***^
Waist_Hip (WHR)	<0.0001	1.762 (1.676–1.852)	<0.0001	1.423 (1.34–1.51)	0.652	0.652	Waist_Hip + TG	0.711	0.725^***^
Pelvic_Hip	<0.0001	1.571 (1.496–1.649)	<0.0001	1.204 (1.136–1.275)	0.628	0.628	Pelvic_Hip + TG	0.703	0.72^***^
Forehead_Waist	<0.0001	0.561 (0.533–0.589)	<0.0001	0.674 (0.636–0.714)	0.657	0.657	Forehead_Waist + TG	0.714	0.726^***^
Neck_Waist	<0.0001	0.703 (0.671-0.737)	<0.0001	0.851 (0.807-0.898)	0.6	0.6	Neck_Waist + TG	0.695	0.716^***^
Forehead_Rib	<0.0001	0.526 (0.5-0.554)	<0.0001	0.634 (0.597-0.672)	0.673	0.673	Forehead_Rib + TG	0.722	0.73^***^
Forehead_Chest	<0.0001	0.556 (0.529-0.585)	<0.0001	0.678 (0.641-0.716)	0.66	0.66	Forehead_Chest + TG	0.718	0.727^***^
WHtR	<0.0001	1.493 (1.422-1.566)	<0.0001	1.439 (1.366-1.515)	0.616	0.616	WHtR + TG	0.699	0.718^***^

Adjustments for age and site (investigation site), *OR* odds ratio, *CI* confidence interval, *AUC* area under the receiver operating characteristic curve, *NB* naïve Bayes, *LR* logistic regression

Significant difference between a single anthropometric index and phenotype on AUCs determined by LR, ^***^ *p* = <0.00001

^a^ There were 311 women with the HW phenotype in the non-hypo-HDL cholesterolemia group (*n* = 3654) and 1173 in the hypo-HDL cholesterolemia group (*n* = 4007)

**Table 4:** Analysis of the associations and powers of models of the HW phenotype and all phenotypes consisting of an individual index with/without the TG level to predict hyper-LDL cholesterolemia

Variable	Crude		Adjustment		AUC		Phenotype	AUC	
	p	OR (95 % CI)	p	OR (95 % CI)	NB	LR		NB	LR
HW phenotype^a^	<0.0001	1.826 (1.541–2.164)	<0.0001	1.459 (1.222–1.741)					
TG	<0.0001	1.324 (1.246–1.406)	<0.0001	1.236 (1.159–1.318)	0.598	0.638			
Age	<0.0001	1.659 (1.525–1.805)			0.653	0.636	Age + TG	0.662	0.65
Weight	<0.0001	1.231 (1.145–1.324)	<0.0001	1.244 (1.154–1.341)	0.57	0.571	Weight + TG	0.603	0.629^***^
BMI	<0.0001	1.418 (1.32–1.524)	<0.0001	1.27 (1.176–1.373)	0.618	0.618	BMI + TG	0.632	0.644^**^
NeckC	0.0004	1.143 (1.062–1.23)	0.0004	1.157 (1.068–1.253)	0.539	0.542	NeckC + TG	0.591	0.631^***^
ChestC	<0.0001	1.404 (1.304–1.511)	<0.0001	1.325 (1.222–1.436)	0.605	0.606	ChestC + TG	0.62	0.634^***^
RibC	<0.0001	1.389 (1.292–1.494)	<0.0001	1.282 (1.179–1.393)	0.607	0.608	RibC + TG	0.622	0.635^***^
WaistC (WC)	<0.0001	1.397 (1.297–1.505)	<0.0001	1.242 (1.142–1.35)	0.609	0.606	WaistC + TG	0.623	0.634^***^
HipC	0.0010	1.132 (1.051–1.218)	0.0005	1.147 (1.061–1.24)	0.534	0.54	HipC + TG	0.599	0.63^***^
Neck_Hip	0.7273	1.013 (0.94–1.093)	0.9135	1.005 (0.925–1.091)	0.484	0.49	Neck_Hip + TG	0.593	0.636^***^
Axillary_Hip	<0.0001	1.258 (1.168–1.355)	<0.0001	1.197 (1.104–1.298)	0.57	0.572	Axillary_Hip + TG	0.6	0.623^***^
Chest_Hip	<0.0001	1.442 (1.338–1.554)	<0.0001	1.311 (1.205–1.426)	0.611	0.611	Chest_Hip + TG	0.622	0.635^***^
Rib_Hip	<0.0001	1.438 (1.334–1.55)	<0.0001	1.283 (1.17–1.405)	0.611	0.613	Rib_Hip + TG	0.622	0.636^***^
Waist_Hip (WHR)	<0.0001	1.464 (1.358–1.579)	<0.0001	1.233 (1.126–1.35)	0.613	0.614	Waist_Hip + TG	0.623	0.636^***^
Pelvic_Hip	<0.0001	1.418 (1.311–1.533)	0.0122	1.124 (1.026–1.232)	0.595	0.597	Pelvic_Hip + TG	0.61	0.629^***^
Forehead_Waist	<0.0001	0.652 (0.6–0.708)	<0.0001	0.767 (0.698–0.843)	0.618	0.616	Forehead_Waist + TG	0.626	0.636^***^
Neck_Waist	<0.0001	0.685 (0.632–0.743)	<0.0001	0.823 (0.753-0.9)	0.604	0.603	Neck_Waist + TG	0.618	0.634^***^
Forehead_Rib	<0.0001	0.655 (0.604–0.709)	<0.0001	0.741 (0.675-0.814)	0.619	0.618	Forehead_Rib + TG	0.628	0.637^***^
Forehead_Chest	<0.0001	0.651 (0.601–0.706)	<0.0001	0.728 (0.665-0.796)	0.617	0.619	Forehead_Chest + TG	0.626	0.637^***^
WHtR	<0.0001	1.334 (1.242–1.434)	<0.0001	1.264 (1.172-1.363)	0.598	0.598	WHtR + TG	0.619	0.635^***^

Adjustments for age and site (investigation site), *OR* odds ratio, *CI* confidence interval, *AUC* area under the receiver operating characteristic curve, *NB* naïve Bayes, *LR* logistic regression

Significant difference between a single anthropometric index and phenotype on AUCs determined by LR, ^**^ *p* = <0.0001, and ^***^ *p* = <0.00001

^a^ There were 1268 women with the HW phenotype in the non-hyper-LDL cholesterolemia group (*n* = 6918) and 216 in the hyper-LDL cholesterolemia group (*n* = 743)

The HW phenotype was the most strongly associated with the LDL level compared with the other single indices (*p* < 0.0001, OR = 1.826 [1.541-2.164], adjusted OR = 1.459 [1.222-1.741]); however, this phenotype was less strongly associated with the LDL level compared with the TC and HDL levels, SBP, and DBP. Regarding WC and the TG level, the association between the TG level and the LDL level (*p* < 0.0001, OR = 1.324 [1.246-1.406], adjusted OR = 1.236 [1.159-1.318]) was similar to that between WC and the LDL level (*p* < 0.0001, OR = 1.397 [1.297-1.505], adjusted OR = 1.242 [1.142-1.35]). Among all phenotypes, the age + TG phenotype was the strongest predictor of the LDL level (AUC by NB = 0.662, AUC by LR = 0.65).

### SBP and DBP

Tables 5 and 6 list the experimental results for the associations of the HW phenotype with SBP and DBP, as well as the powers of the models of all index/TG combinations to predict hypertension. The HW phenotype had the strongest association with SBP among all variables (*p* < 0.0001, OR = 2.362 [2.029-2.749], adjusted OR = 1.646 [1.397-1.938]). Among all single indices, age was the most strongly associated with SBP (*p* < 0.0001, OR = 2.345 [2.152-2.555]) after HW and was the best predictor of SBP (AUC = 0.707). WC (*p* < 0.0001, OR = 1.677 [1.564-1.797], adjusted OR = 1.334 [1.233-1.444]) was more strongly associated with SBP than with the TG level (*p* < 0.0001, OR = 1.377 [1.3-1.459], adjusted OR = 1.24 [1.165-1.321]). WC (AUC = 0.653) was also a stronger predictor than the TG level (AUC by NB = 0.62, AUC by LR = 0.631). Age + TG was the best predictor of SBP status (AUC by NB = 0.713, AUC by LR = 0.718).

**Table 5:** Analysis of the associations and powers of models of the HW phenotype and all phenotypes consisting of an individual index with/without the TG level to predict SBP

Variable	Crude		Adjustment		AUC		Phenotype	AUC	
	p	OR (95 % CI)	p	OR (95 % CI)	NB	LR		NB	LR
HW phenotype^a^	<0.0001	2.362 (2.029–2.749)	<0.0001	1.646 (1.397–1.938)					
TG	<0.0001	1.377 (1.3–1.459)	<0.0001	1.24 (1.165–1.321)	0.62	0.631			
Age	<0.0001	2.345 (2.152–2.555)			0.707	0.707	Age + TG	0.713	0.718
Weight	<0.0001	1.153 (1.078–1.234)	<0.0001	1.271 (1.183–1.366)	0.547	0.541	Weight + TG	0.621	0.627^***^
BMI	<0.0001	1.483 (1.388–1.585)	<0.0001	1.364 (1.267–1.468)	0.62	0.621	BMI + TG	0.644	0.649^***^
NeckC	<0.0001	1.449 (1.356–1.548)	<0.0001	1.309 (1.216–1.409)	0.616	0.616	NeckC + TG	0.641	0.648^*^
ChestC	<0.0001	1.529 (1.428–1.637)	<0.0001	1.355 (1.255–1.463)	0.627	0.627	ChestC + TG	0.645	0.65^***^
RibC	<0.0001	1.714 (1.601–1.834)	<0.0001	1.385 (1.28–1.498)	0.665	0.665	RibC + TG	0.672	0.677^*^
WaistC (WC)	<0.0001	1.677 (1.564–1.797)	<0.0001	1.334 (1.233–1.444)	0.653	0.653	WaistC + TG	0.61	0.626^***^
HipC	<0.0001	1.178 (1.101–1.26)	<0.0001	1.187 (1.104–1.278)	0.54	0.543	HipC + TG	0.623	0.637^***^
Neck_Hip	<0.0001	1.269 (1.188–1.356)	0.0043	1.114 (1.035–1.2)	0.575	0.575	Neck_Hip + TG	0.626	0.635^***^
Axillary_Hip	<0.0001	1.356 (1.267–1.452)	<0.0001	1.211 (1.122–1.307)	0.591	0.592	Axillary_Hip + TG	0.647	0.651^***^
Chest_Hip	<0.0001	1.563 (1.459–1.676)	<0.0001	1.295 (1.196–1.402)	0.629	0.629	Chest_Hip + TG	0.647	0.651^***^
Rib_Hip	<0.0001	1.888 (1.758–2.029)	<0.0001	1.388 (1.273–1.513)	0.685	0.685	Rib_Hip + TG	0.687	0.692^*^
Waist_Hip (WHR)	<0.0001	1.835 (1.707–1.972)	<0.0001	1.327 (1.217–1.446)	0.67	0.67	Waist_Hip + TG	0.675	0.681^***^
Pelvic_Hip	<0.0001	1.649 (1.531–1.777)	<0.0001	1.25 (1.142–1.369)	0.639	0.641	Pelvic_Hip + TG	0.657	0.663^***^
Forehead_Waist	<0.0001	0.542 (0.5–0.586)	<0.0001	0.717 (0.655–0.784)	0.663	0.663	Forehead_Waist + TG	0.666	0.673^***^
Neck_Waist	<0.0001	0.678 (0.63–0.731)	0.0007	0.866 (0.796–0.941)	0.609	0.609	Neck_Waist + TG	0.633	0.641^***^
Forehead_Rib	<0.0001	0.519 (0.48–0.561)	<0.0001	0.677 (0.619–0.742)	0.676	0.676	Forehead_Rib + TG	0.677	0.683^**^
Forehead_Chest	<0.0001	0.602 (0.559–0.649)	<0.0001	0.712 (0.654–0.776)	0.639	0.639	Forehead_Chest + TG	0.65	0.654^***^
WHtR	<0.0001	1.318 (1.233–1.408)	<0.0001	1.322 (1.23–1.421)	0.581	0.584	WHtR + TG	0.626	0.632^***^

Adjustments for age and site (investigation site), *OR* odds ratio, *CI* confidence interval, *AUC* area under the receiver operating characteristic curve, *NB* naïve Bayes, *LR* logistic regression

Significant difference between a single anthropometric index and phenotype on AUCs determined by LR, ^*^ *p* = <0.01, ^**^ *p* = <0.0001, and ^***^ *p* = <0.00001

^a^ There were 1182 women with the HW phenotype in the normal SBP group (*n* = 6756) and 302 in the high SBP group (*n* = 905)

**Table 6:** Analysis of the associations and powers of models of the HW phenotype and all phenotypes consisting of an individual index with/without the TG level to predict DBP

Variable	Crude		Adjustment		AUC		Phenotype	AUC	
	p	OR (95 % CI)	p	OR (95 % CI)	NB	LR		NB	LR
HW phenotype^a^	<0.0001	2.174 (1.877–2.517)	<0.0001	1.946 (1.664–2.276)					
TG	<0.0001	1.276 (1.207–1.35)	<0.0001	1.236 (1.164–1.313)	0.585	0.591			
Age	<0.0001	1.331 (1.242–1.426)			0.572	0.572	Age + TG	0.602	0.6^*^
Weight	<0.0001	1.368 (1.284–1.457)	<0.0001	1.425 (1.335–1.521)	0.59	0.593	Weight + TG	0.609	0.614^**^
BMI	<0.0001	1.48 (1.389–1.577)	<0.0001	1.492 (1.393–1.598)	0.618	0.618	BMI + TG	0.625	0.627
NeckC	<0.0001	1.514 (1.42–1.613)	<0.0001	1.387 (1.296–1.485)	0.628	0.628	NeckC + TG	0.638	0.64
ChestC	<0.0001	1.56 (1.461–1.665)	<0.0001	1.516 (1.411–1.629)	0.63	0.63	ChestC + TG	0.634	0.637^*^
RibC	<0.0001	1.57 (1.472–1.674)	<0.0001	1.451 (1.35–1.56)	0.637	0.637	RibC + TG	0.638	0.642
WaistC (WC)	<0.0001	1.546 (1.448–1.651)	<0.0001	1.459 (1.355–1.57)	0.629	0.629	WaistC + TG	0.633	0.637^*^
HipC	<0.0001	1.377 (1.293–1.467)	<0.0001	1.358 (1.27–1.451)	0.589	0.591	HipC + TG	0.61	0.615^*^
Neck_Hip	0.0002	1.128 (1.058–1.203)	0.5271	1.023 (0.954–1.097)	0.535	0.536	Neck_Hip + TG	0.59	0.596^***^
Axillary_Hip	<0.0001	1.222 (1.145–1.304)	<0.0001	1.183 (1.102–1.269)	0.56	0.562	Axillary_Hip + TG	0.596	0.599^***^
Chest_Hip	<0.0001	1.344 (1.259–1.435)	<0.0001	1.289 (1.196–1.389)	0.586	0.587	Chest_Hip + TG	0.606	0.607^***^
Rib_Hip	<0.0001	1.421 (1.33–1.517)	<0.0001	1.272 (1.174–1.379)	0.605	0.606	Rib_Hip + TG	0.618	0.618^*^
Waist_Hip (WHR)	<0.0001	1.406 (1.316–1.503)	<0.0001	1.31 (1.207–1.421)	0.601	0.602	Waist_Hip + TG	0.615	0.617^**^
Pelvic_Hip	<0.0001	1.265 (1.182–1.354)	<0.0001	1.208 (1.112–1.313)	0.559	0.563	Pelvic_Hip + TG	0.591	0.596^***^
Forehead_Waist	<0.0001	0.643 (0.599–0.691)	<0.0001	0.66 (0.607–0.718)	0.621	0.622	Forehead_Waist + TG	0.625	0.629^*^
Neck_Waist	<0.0001	0.788 (0.736–0.844)	<0.0001	0.814 (0.753–0.879)	0.568	0.569	Neck_Waist + TG	0.595	0.599^***^
Forehead_Rib	<0.0001	0.629 (0.586–0.675)	<0.0001	0.658 (0.606–0.715)	0.628	0.628	Forehead_Rib + TG	0.63	0.633^*^
Forehead_Chest	<0.0001	0.648 (0.604–0.696)	<0.0001	0.643 (0.593–0.696)	0.62	0.62	Forehead_Chest + TG	0.624	0.627^*^
WHtR	<0.0001	1.44 (1.352–1.534)	<0.0001	1.466 (1.373–1.567)	0.609	0.61	WHtR + TG	0.62	0.623^*^

Adjustments for age and site (investigation site), *OR* odds ratio, *CI* confidence interval, *AUC* area under the receiver operating characteristic curve, *NB* naïve Bayes, *LR* logistic regression

Significant difference between a single anthropometric index and phenotype on AUCs determined by LR, ^*^ *p* = <0.01, ^**^ *p* = <0.0001, and ^***^ *p* = <0.00001

^a^ There were 1163 women with the HW phenotype in the normal DBP group (*n* = 6644) and 321 in the high DBP group (*n* = 1017)

The HW phenotype was the most strongly associated with DBP compared with all variables, and after adjustments for site and age, this association remained the strongest (*p* < 0.0001, OR = 2.174 [1.877-2.517], adjusted OR = 1.946 [1.664-2.276]). WC was more strongly associated with DBP (*p* < 0.0001, OR = 1.546 [1.448-1.651], adjusted OR = 1.459 [1.355-1.57]) than with the TG level (*p* < 0.0001, OR = 1.276 [1.207-1.35], adjusted OR = 1.236 [1.164-1.313]), and WC (AUC = 0.629) was a stronger predictor than the TG level (AUC by NB = 0.585, AUC by LR = 0.591). Among the phenotypes, RibC + TG (AUC by NB = 0.638, AUC by LR = 0.642) and NeckC + TG (AUC by NB = 0.638, AUC by LR = 0.64) were the strongest indicators of DBP status; however, the addition of the TG level to WC added very little predictive power.

## Discussion

This study, which was conducted on a population of Korean women, has identified a strong association between the HW phenotype and metabolic abnormalities. Furthermore, the results have shown that the best phenotype for identifying metabolic abnormalities may differ according to the metabolic factor of interest.

Many studies have indicated that the HW phenotype is closely associated with metabolic abnormalities, and it is a simple, inexpensive and accurate predictor of these abnormalities [1–16, 23, 27, 30, 38]. For example, Lemieux et al. [11] indicated that the combination of WC and the TG level constitutes a simple and inexpensive indicator of the risk of atherogenic metabolic abnormalities and CAD in Canadian men. Irving et al. [14] reported that compared with women with MS but without HW, those with both MS and HW tended to have a lower total HDL cholesterol level and higher abdominal visceral fat mass and insulin, TC, non-HDL cholesterol, and VLDL cholesterol levels. They argued that in obese women with MS and HW, the HW aggravates cardiometabolic abnormalities and insulin resistance. Esmaillzadeh et al. [4] proposed HW as a simple and efficient predictor of metabolic abnormalities and MS in adolescents. Additionally, Zhang et al. [43] found that in Chinese men and women, HW and the visceral adiposity index score (defined by the HDL cholesterol and TG levels, WC, and BMI) were strongly associated with coronary heart disease. These authors suggested that HW was an indicator of the risk of coronary heart disease in Asian individuals of normal weight with visceral adiposity. de Graaf et al. [6] indicated that individuals with type-2 diabetes and HW tended to have an increased risk of coronary artery disease and abnormal blood lipid profiles compared with those without HW and that HW was a practical clinical indicator of coronary artery disease risk in subjects with type-2 diabetes. Hiura et al. [5] tested the utility of HW as a predictor of CVD risk in indigenous Australian women and found that HW was associated with an elevated TC-to-HDL ratio and higher TC, LDL, and TG levels. These authors also suggested that HW was a good indicator of high plasma insulin and apolipoprotein B levels and thus, of cardiovascular risk. Amini et al. [3] reported that HW was associated with diabetes in men and women and with impaired glucose tolerance in women. Our findings are in support of those of previous studies. We found that among the anthropometric indices, HW was the most strongly associated with the TC, HDL, and LDL levels, SBP, and DBP.

Several studies have demonstrated the superiority of the HW phenotype for detecting metabolic abnormalities in terms of its simplicity, cost-effectiveness, and discriminatory ability compared with the NCEP-ATP III and International Diabetes Federation (IDF) criteria. For example, Zainuddin et al. [15] examined the concordance of the IDF and NCEP-ATP III criteria and HW in evaluating the prevalence of MS in Malaysian men and women and argued that because the three criteria were sufficiently concordant, HW should be used as a primary screening tool to detect MS in developing countries. Blackburn et al. [13] compared the abilities of HW and the NCEP-ATP III and IDF criteria to identify subjects with cardiometabolic risk factors and suggested that HW might have a similar discriminatory power and that it could be used as the primary screening tool to detect subjects at higher risk of cardiometabolic disease. In a Spanish population, Gomez-Huelgas et al. [12] also found that the prevalence of HW was lower than that determined by the MS criteria defined by the NCEP-ATP III and IDF. Individuals with HW had a higher prevalence of hyperglycemia and/or type-2 diabetes compared with those without HW. They suggested that HW is a useful indicator of CVD and type-2 diabetes in young subjects who do not meet the criteria for MS. A study conducted by Gazi et al. [10] has revealed that HW screening is simple and cost-effective for detecting metabolic abnormalities compared with the use of the MS criteria. Blackburn et al. [30] found that Canadian women with HW had more metabolic risk factors and a higher BMI and WC compared with women without HW. They suggested that HW was comparable to the NCEP-ATP III criteria as a tool for identifying Canadian women with cardiometabolic risk factors and coronary artery disease.

Some studies have demonstrated that metabolic abnormalities are more strongly associated with the HW phenotype than with the TG level or WC alone [3, 5, 16, 23, 27]. For example, Després et al. [27] have found that obese subjects with HW can present with strong risk factors for coronary heart disease even without hypertension, hypercholesterolemia, or hyperglycemia. They argued that the combined use of WC and the TG level to identify subjects at high risk of disease might be better compared with the use of WC or the TG level alone. A seven-year longitudinal study of women has demonstrated that WC is a better correlate of visceral adipose tissue mass compared with the WHR. Okosuna and Boltrib [16] found that individuals with HW were at a higher risk of type-2 diabetes compared with those with either hypertriglyceridemia or increased WC alone. They determined that the association of type-2 diabetes with HW was substantially higher in American men and women of African descent compared with Caucasians. Sam et al. [23] indicated that in non-Hispanic white and non-Hispanic black subjects in Chicago, WC alone was not predictive of a higher degree of visceral fat accumulation, even in individuals with type-2 diabetes. They found that the relationship between HW and coronary atherosclerosis may be associated with the proatherogenic lipoprotein changes related to HW and that the combination of the fasting TG level and WC constituted a simple and inexpensive tool to predict high visceral fat mass, CVD and metabolic disease. Our findings are thus consistent with those of these previous studies. We found that all combinations of an anthropometric index + TG were more strongly predictive of the five metabolic components than the single indices alone, with the exception of waist + TC for SBP. Combined phenotypes that include the TG level are thus better predictors of metabolic abnormalities compared with a single anthropometric index.

Our findings demonstrated that age was a stronger predictor of the TC and LDL cholesterol levels and SBP. Additionally, SBP was more strongly associated with age compared with DBP. Our results are consistent with those of previous studies [44–49]. Barbieri et al. [44] have argued that the important indicators of SBP include age, the severity of carotid atherosclerosis, and the insulin resistance syndrome score. Wright et al. [45] have found that age, gender, smoking, and weight are correlated with SBP, whereas anxiety and weight, but not age, are correlated with DBP. Terzolo et al. [46] have documented that age and the midnight cortisol level are associated with SBP, but not with DBP. In a study of the association between blood pressure and age, gender, and BMI and the association between BP and ECG signs in 1851 Inuits in East Greenland, Andersen et al. [47] found that age and BMI were associated with high SBP and that age and gender were associated with high DBP; however, they found that age was more strongly correlated with SBP than with DBP. van Stiphout et al. [48] determined that age, weight, and the parental cholesterol concentration were associated with the TC concentration in a population derived from two regions of the Dutch town of Zoetermeer. Further, Berge and Nordøy [49] argued that age was strongly associated with the TC concentration in a study of the effects of age and menopause and the associations among risk factors of CVD, serum ferritin, and sex hormones in healthy premenopausal and postmenopausal women.

Among metabolic components, such as the TC, HDL, and LDL levels, SBP, and DBP, we found that the HW phenotype was the least strongly associated with the LDL level. One cause of this phenomenon may be the weak association between the LDL level and the anthropometric indices compared with the other components. For example, in a study of the correlations between anthropometric indices and cardiometabolic factors in Hispanics living in Puerto Rico, Palacios et al. [50] showed that the correlations of LDL with WC, BMI, and the WHR and waist-to-height circumference ratio (WHtR) were lower compared with those of LDL with HDL, SBP, DBP, and TC/HDL. In a study of an urban population in eastern India, Prasad et al. [51] found that LDL was very weakly associated with anthropometric indices, such as BMI, WC, and the WHR and WHtR compared with the associations of SBP, DBP, TC, or HDL with the four anthropometric indices. BMI, WC, and the WHR and WHtR were weaker predictors of the LDL level compared with SBP, DBP, TC, or HDL.

For increased validity, the data were analyzed using two statistical methods, NB and LR, and the AUC values of the indices and phenotypes obtained using these two methods were found to be the same or similar for the five metabolic abnormalities. For the TC level (hypercholesterolemia), the differences in the AUC values between NB and LR were approximately ±0.039–0.001 for the individual indices and approximately ±0.048–0.018 for the phenotypes. For the HDL level (hypo-HDL cholesterolemia), the differences in the AUC values between the two statistical methods were approximately the same, with the exceptions of ±0.001 for HipC, Neck_Hip, and Rib_Hip and 0.031 for TG for the individual indices and approximately ±0.029–0.008 for the phenotypes. For the LDL level (hyper-LDL cholesterolemia), the differences in the AUC values were approximately ±0.04–0 for the individual indices and approximately ±0.043–0.009 for the phenotypes. The differences in the AUC values between NB and LR were approximately ±0.01–0 for the individual indices and approximately ±0.016–0.004 for the phenotypes for SBP, and they were approximately ±0.006–0 for both the individual indices and phenotypes for DBP.

Finally, all individual variables were stronger predictors when combined with the TG level compared with the use of each variable alone. Thus, phenotypes can provide better results compared with single anthropometric indices in the prediction of metabolic abnormalities. We think that this is a logical result because it is well known that the TG level, which is included in the various phenotypes in this study, is strongly associated with MS and that it is a factor for this syndrome. Considering the limitations of the study, in the future, we will consider associations among specific and regional characteristics of body fat mass, TG, and MS. We examined several characteristics related to particular body locations in this study. The associations between these specific locations and metabolic abnormalities may differ according to age, gender, ethnic group, and country. In addition, with regard to feature selection techniques, the limitation of this study is that the assessment of phenotypes according to a combination of TG and one anthropometric index may not be useful for improving the prediction of metabolic abnormalities or MS. Feature selection was not used in our experiments. We simply examined the combination of a single anthropometric index and the TG level, for example, age + TG, weight + TG, BMI + TG, etc., because the aim of this study was to assess phenotypes consisting of one individual index and the TG level. However, the use of such combinations may result in the omission of important feature combinations or optimal feature subsets. In addition, models constructed using various feature selection techniques and search strategies may achieve increased predictive power for metabolic abnormalities or MS. For example, a top-down strategy (backward elimination) begins with all features and then sequentially eliminates irrelevant features, and a bottom-up strategy (forward selection) begins with an empty feature set or the most important feature and iteratively adds features [52–54]. Further study is needed to identify optimal feature subsets using various feature selection techniques and search strategies for the improved prediction of metabolic abnormalities and MS.

## Conclusions

A large number of studies have suggested that the HW phenotype is strongly associated with metabolic abnormalities, CVD, and diabetes; however, to date, no study has reported the predictive power of the TG level combined with WC and with single anthropometric indices. In this study, we demonstrated an association between the HW phenotype and metabolic abnormalities, in addition to the power of various phenotypes to predict metabolic abnormalities in Korean women. Our findings suggest that although the HW phenotype is the most strongly associated with metabolic abnormalities, the best predictive phenotypes consisting of an individual index and the TG level may differ according to the metabolic factors present. Phenotypes can provide better predictive power compared with single anthropometric indices in the identification of metabolic abnormalities. To our knowledge, this is the first study that has assessed the predictive power of phenotypes consisting of the TG level and individual anthropometric indices to identify subjects with metabolic abnormalities.

## Abbreviations

AUC: the area under the receiver operating characteristic curve
DBP: diastolic blood pressure
HDL: high-density lipoprotein cholesterol
HW: hypertriglyceridemic waist phenotype
LDL: low-density lipoprotein cholesterol
LR: logistic regression
NB: Naïve Bayes algorithm
SBP: systolic blood pressure
TC: total cholesterol
TG: triglyceride
